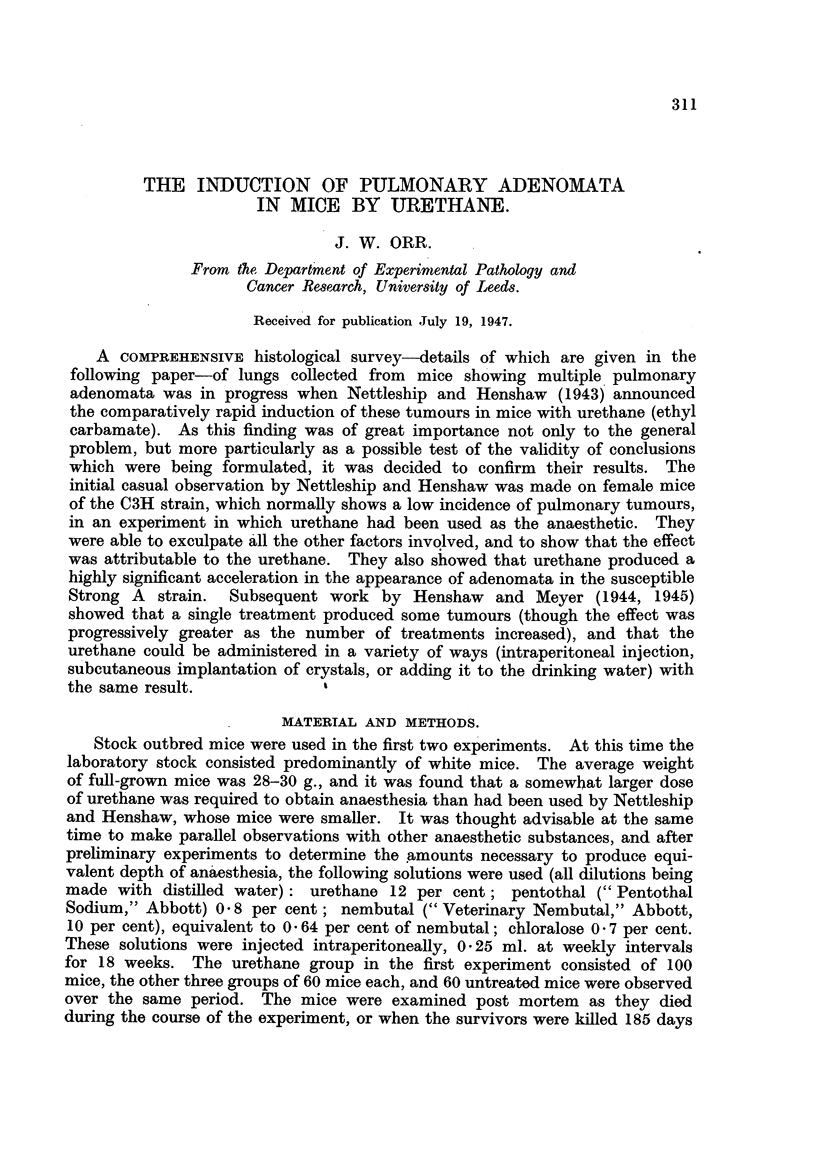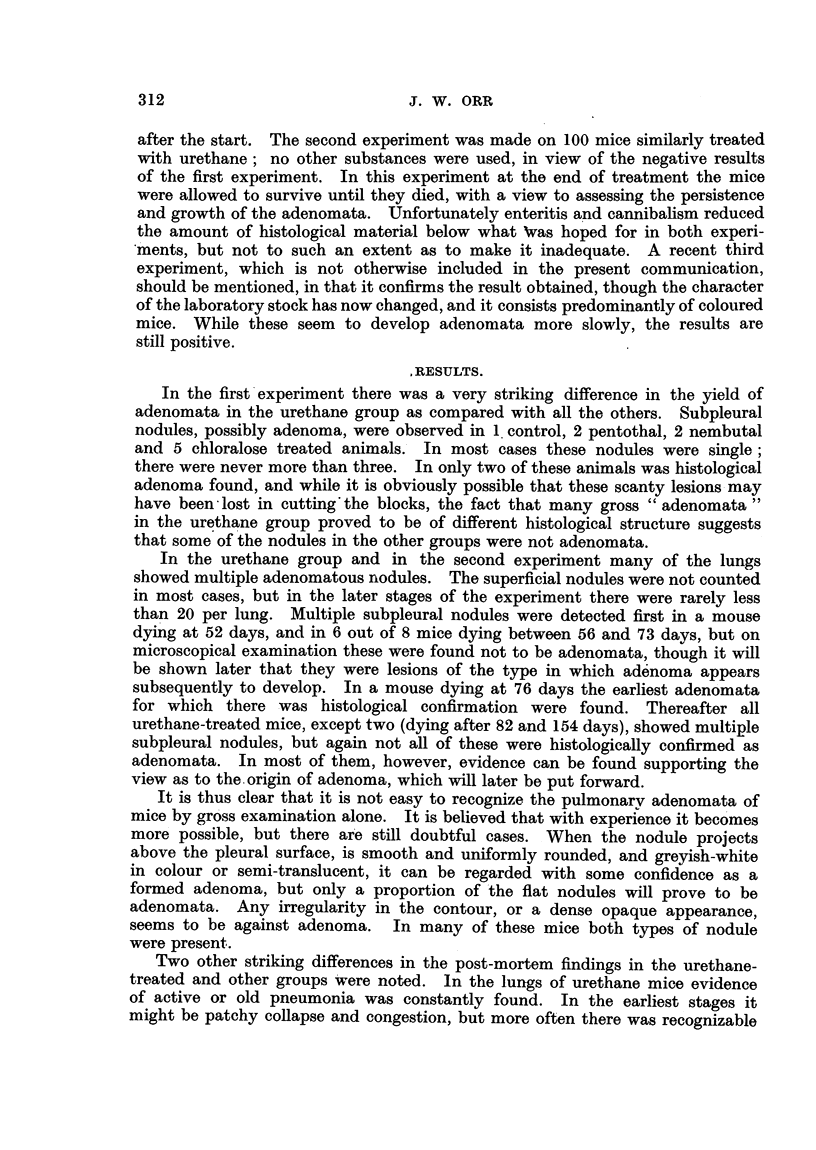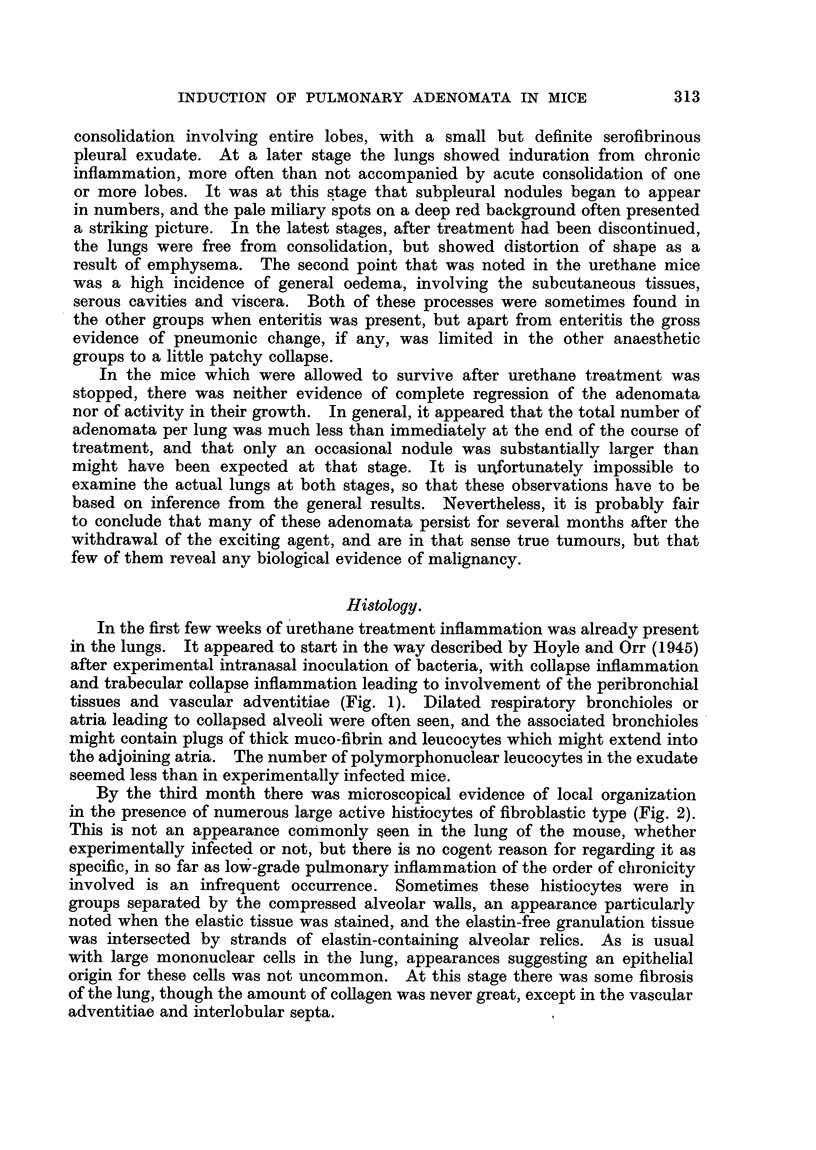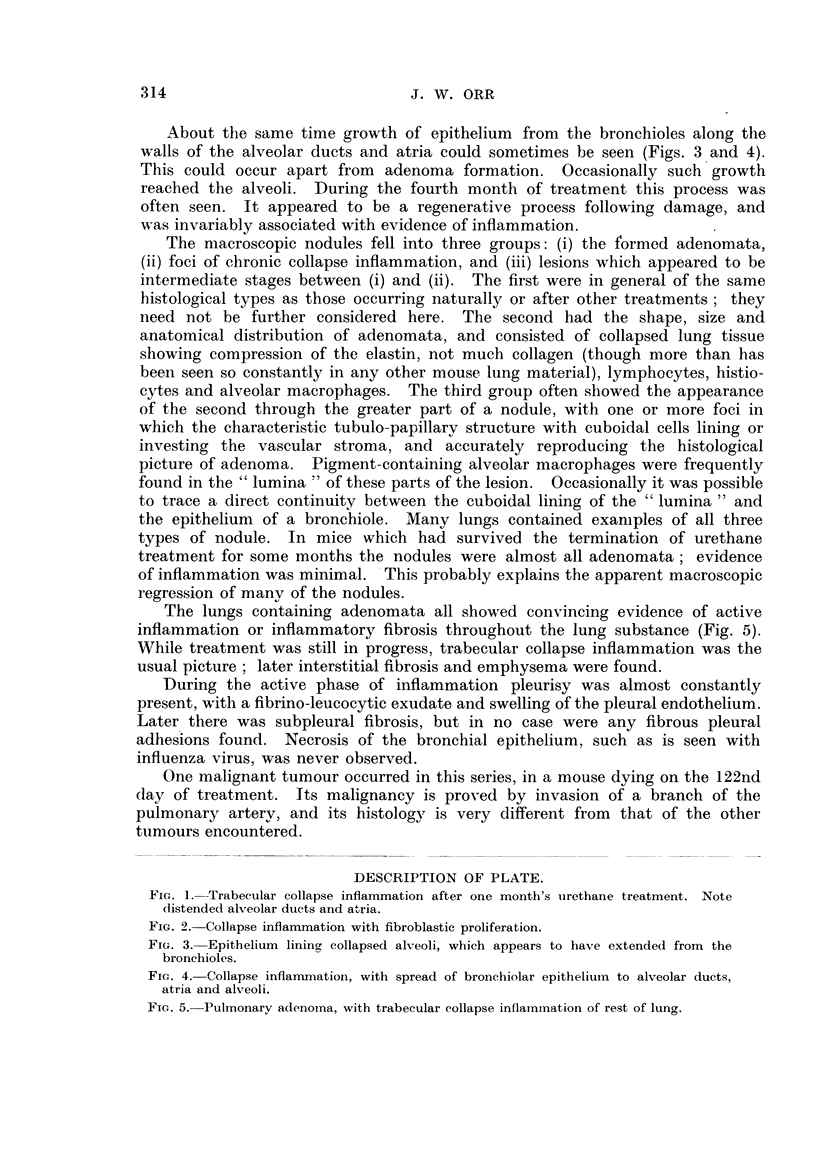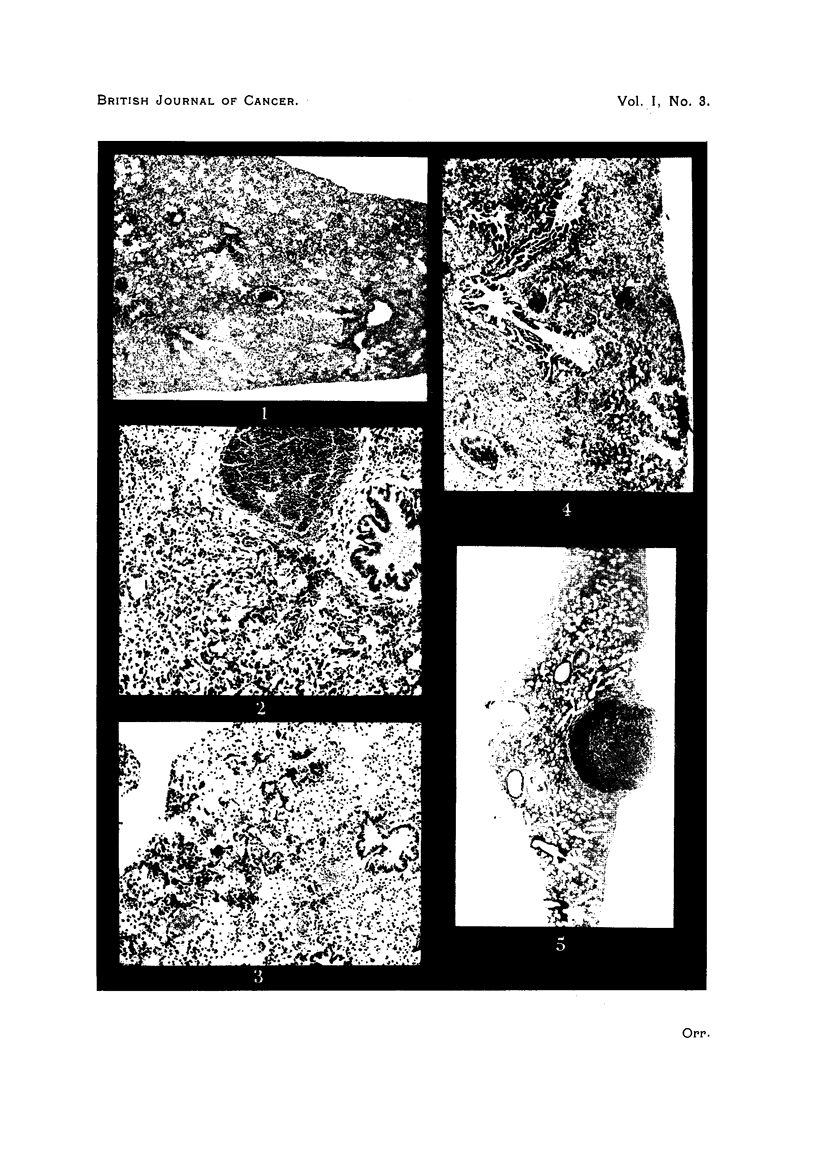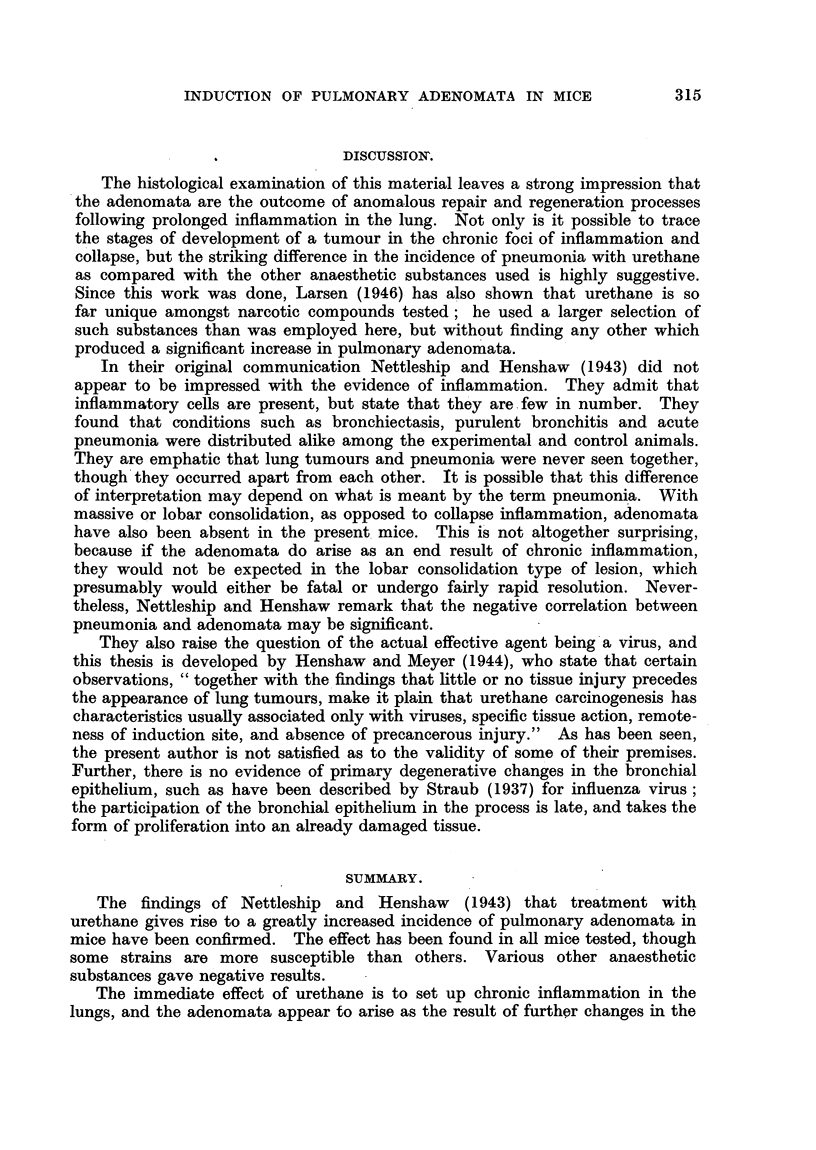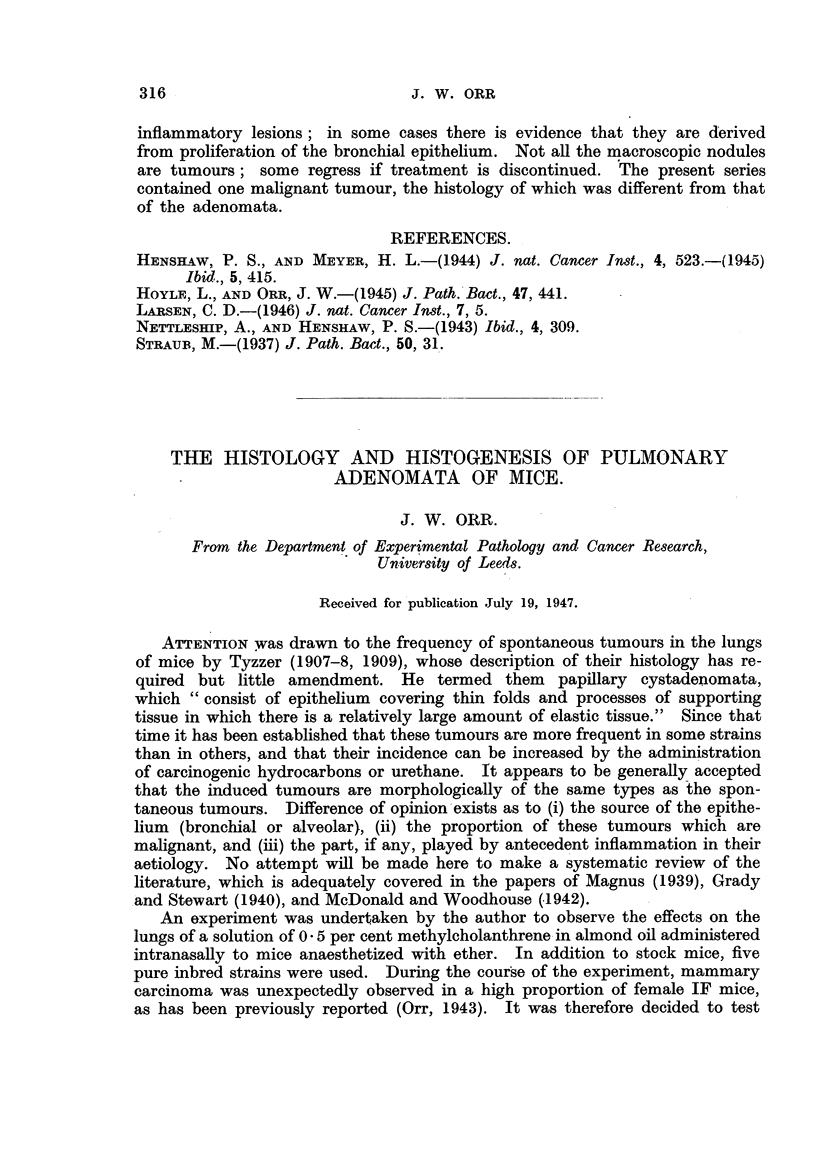# The Induction of Pulmonary Adenomata in Mice by Urethane

**DOI:** 10.1038/bjc.1947.26

**Published:** 1947-09

**Authors:** J. W. Orr

## Abstract

**Images:**


### THE INDUCTION OF PULMONARY ADENOMATA IN MICE BY URETHANE.

J. W. ORR.

*From the Department of Experimental Pathology and Cancer Research, University of Leeds.*

Received for publication July 19, 1947.

A COMPREHENSIVE histological survey—details of which are given in the following paper—of lungs collected from mice showing multiple pulmonary adenomata was in progress when Nettleship and Henshaw (1943) announced the comparatively rapid induction of these tumours in mice with urethane (ethyl carbamate). As this finding was of great importance not only to the general problem, but more particularly as a possible test of the validity of conclusions which were being formulated, it was decided to confirm their results. The initial casual observation by Nettleship and Henshaw was made on female mice of the C3H strain, which normally shows a low incidence of pulmonary tumours, in an experiment in which urethane had been used as the anaesthetic. They were able to exculpate all the other factors involved, and to show that the effect was attributable to the urethane. They also showed that urethane produced a highly significant acceleration in the appearance of adenomata in the susceptible Strong A strain. Subsequent work by Henshaw and Meyer (1944, 1945) showed that a single treatment produced some tumours (though the effect was progressively greater as the number of treatments increased), and that the urethane could be administered in a variety of ways (intraperitoneal injection, subcutaneous implantation of crystals, or adding it to the drinking water) with the same result.

### MATERIAL AND METHODS.

Stock outbred mice were used in the first two experiments. At this time the laboratory stock consisted predominantly of white mice. The average weight of full-grown mice was 28–30 g., and it was found that a somewhat larger dose of urethane was required to obtain anaesthesia than had been used by Nettleship and Henshaw, whose mice were smaller. It was thought advisable at the same time to make parallel observations with other anaesthetic substances, and after preliminary experiments to determine the amounts necessary to produce equivalent depth of anaesthesia, the following solutions were used (all dilutions being made with distilled water): urethane 12 per cent; pentothal ("Pentothal Sodium," Abbott) 0·8 per cent; nembutal ("Veterinary Nembutal," Abbott, 10 per cent), equivalent to 0·64 per cent of nembutal; chloralose 0·7 per cent. These solutions were injected intraperitoneally, 0·25 ml. at weekly intervals for 18 weeks. The urethane group in the first experiment consisted of 100 mice, the other three groups of 60 mice each, and 60 untreated mice were observed over the same period. The mice were examined post mortem as they died during the course of the experiment, or when the survivors were killed 185 days after the start. The second experiment was made on 100 mice similarly treated with urethane ; no other substances were used, in view of the negative results of the first experiment. In this experiment at the end of treatment the mice were allowed to survive until they died, with a view to assessing the persistence and growth of the adenomata. Unfortunately enteritis and cannibalism reduced the amount of histological material below what was hoped for in both experiments, but not to such an extent as to make it inadequate. A recent third experiment, which is not otherwise included in the present communication, should be mentioned, in that it confirms the result obtained, though the character of the laboratory stock has now changed, and it consists predominantly of coloured mice. While these seem to develop adenomata more slowly, the results are still positive.

### RESULTS.

In the first experiment there was a very striking difference in the yield of adenomata in the urethane group as compared with all the others. Subpleural nodules, possibly adenoma, were observed in 1 control, 2 pentothal, 2 nembutal and 5 chloralose treated animals. In most cases these nodules were single ; there were never more than three. In only two of these animals was histological adenoma found, and while it is obviously possible that these scanty lesions may have been lost in cutting the blocks, the fact that many gross " adenomata " in the urethane group proved to be of different histological structure suggests that some of the nodules in the other groups were not adenomata.

In the urethane group and in the second experiment many of the lungs showed multiple adenomatous nodules. The superficial nodules were not counted in most cases, but in the later stages of the experiment there were rarely less than 20 per lung. Multiple subpleural nodules were detected first in a mouse dying at 52 days, and in 6 out of 8 mice dying between 56 and 73 days, but on microscopical examination these were found not to be adenomata, though it will be shown later that they were lesions of the type in which adenoma appears subsequently to develop. In a mouse dying at 76 days the earliest adenomata for which there was histological confirmation were found. Thereafter all urethane-treated mice, except two (dying after 82 and 154 days), showed multiple subpleural nodules, but again not all of these were histologically confirmed as adenomata. In most of them, however, evidence can be found supporting the view as to the origin of adenoma, which will later be put forward.

It is thus clear that it is not easy to recognize the pulmonary adenomata of mice by gross examination alone. It is believed that with experience it becomes more possible, but there are still doubtful cases. When the nodule projects above the pleural surface, is smooth and uniformly rounded, and greyish-white in colour or semi-translucent, it can be regarded with some confidence as a formed adenoma, but only a proportion of the flat nodules will prove to be adenomata. Any irregularity in the contour, or a dense opaque appearance, seems to be against adenoma. In many of these mice both types of nodule were present.

Two other striking differences in the post-mortem findings in the urethane-treated and other groups were noted. In the lungs of urethane mice evidence of active or old pneumonia was constantly found. In the earliest stages it might be patchy collapse and congestion, but more often there was recognizable consolidation involving entire lobes, with a small but definite serofibrinous pleural exudate. At a later stage the lungs showed induration from chronic inflammation, more often than not accompanied by acute consolidation of one or more lobes. It was at this stage that subpleural nodules began to appear in numbers, and the pale miliary spots on a deep red background often presented a striking picture. In the latest stages, after treatment had been discontinued, the lungs were free from consolidation, but showed distortion of shape as a result of emphysema. The second point that was noted in the urethane mice was a high incidence of general oedema, involving the subcutaneous tissues, serous cavities and viscera. Both of these processes were sometimes found in the other groups when enteritis was present, but apart from enteritis the gross evidence of pneumonic change, if any, was limited in the other anaesthetic groups to a little patchy collapse.

In the mice which were allowed to survive after urethane treatment was stopped, there was neither evidence of complete regression of the adenomata nor of activity in their growth. In general, it appeared that the total number of adenomata per lung was much less than immediately at the end of the course of treatment, and that only an occasional nodule was substantially larger than might have been expected at that stage. It is unfortunately impossible to examine the actual lungs at both stages, so that these observations have to be based on inference from the general results. Nevertheless, it is probably fair to conclude that many of these adenomata persist for several months after the withdrawal of the exciting agent, and are in that sense true tumours, but that few of them reveal any biological evidence of malignancy.

*Histology.*

In the first few weeks of urethane treatment inflammation was already present in the lungs. It appeared to start in the way described by Hoyle and Orr (1945) after experimental intranasal inoculation of bacteria, with collapse inflammation and trabecular collapse inflammation leading to involvement of the peribronchial tissues and vascular adventitiae (Fig. 1). Dilated respiratory bronchioles or atria leading to collapsed alveoli were often seen, and the associated bronchioles might contain plugs of thick muco-fibrin and leucocytes which might extend into the adjoining atria. The number of polymorphonuclear leucocytes in the exudate seemed less than in experimentally infected mice.

By the third month there was microscopical evidence of local organization in the presence of numerous large active histiocytes of fibroblastic type (Fig. 2). This is not an appearance commonly seen in the lung of the mouse, whether experimentally infected or not, but there is no cogent reason for regarding it as specific, in so far as low-grade pulmonary inflammation of the order of chronicity involved is an infrequent occurrence. Sometimes these histiocytes were in groups separated by the compressed alveolar walls, an appearance particularly noted when the elastic tissue was stained, and the elastin-free granulation tissue was intersected by strands of elastin-containing alveolar relics. As is usual with large mononuclear cells in the lung, appearances suggesting an epithelial origin for these cells was not uncommon. At this stage there was some fibrosis of the lung, though the amount of collagen was never great, except in the vascular adventitiae and interlobular septa.

About the same time growth of epithelium from the bronchioles along the walls of the alveolar ducts and atria could sometimes be seen (Figs. 3 and 4). This could occur apart from adenoma formation. Occasionally such growth reached the alveoli. During the fourth month of treatment this process was often seen. It appeared to be a regenerative process following damage, and was invariably associated with evidence of inflammation.

The macroscopic nodules fell into three groups: (i) the formed adenomata, (ii) foci of chronic collapse inflammation, and (iii) lesions which appeared to be intermediate stages between (i) and (ii). The first were in general of the same histological types as those occurring naturally or after other treatments ; they need not be further considered here. The second had the shape, size and anatomical distribution of adenomata, and consisted of collapsed lung tissue showing compression of the elastin, not much collagen (though more than has been seen so constantly in any other mouse lung material), lymphocytes, histiocytes and alveolar macrophages. The third group often showed the appearance of the second through the greater part of a nodule, with one or more foci in which the characteristic tubulo-papillary structure with cuboidal cells lining or investing the vascular stroma, and accurately reproducing the histological picture of adenoma. Pigment-containing alveolar macrophages were frequently found in the " lumina " of these parts of the lesion. Occasionally it was possible to trace a direct continuity between the cuboidal lining of the " lumina " and the epithelium of a bronchiole. Many lungs contained examples of all three types of nodule. In mice which had survived the termination of urethane treatment for some months the nodules were almost all adenomata ; evidence of inflammation was minimal. This probably explains the apparent macroscopic regression of many of the nodules.

The lungs containing adenomata all showed convincing evidence of active inflammation or inflammatory fibrosis throughout the lung substance (Fig. 5). While treatment was still in progress, trabecular collapse inflammation was the usual picture ; later interstitial fibrosis and emphysema were found.

During the active phase of inflammation pleurisy was almost constantly present, with a fibrino-leucocytic exudate and swelling of the pleural endothelium. Later there was subpleural fibrosis, but in no case were any fibrous pleural adhesions found. Necrosis of the bronchial epithelium, such as is seen with influenza virus, was never observed.

One malignant tumour occurred in this series, in a mouse dying on the 122nd day of treatment. Its malignancy is proved by invasion of a branch of the pulmonary artery, and its histology is very different from that of the other tumours encountered.

---

DESCRIPTION OF PLATE.

FIG. 1.—Trabecular collapse inflammation after one month's urethane treatment. Note distended alveolar ducts and atria.

FIG. 2.—Collapse inflammation with fibroblastic proliferation.

FIG. 3.—Epithelium lining collapsed alveoli, which appears to have extended from the bronchioles.

FIG. 4.—Collapse inflammation, with spread of bronchiolar epithelium to alveolar ducts, atria and alveoli.

FIG. 5.—Pulmonary adenoma, with trabecular collapse inflammation of rest of lung.

Orr.

### DISCUSSION.

The histological examination of this material leaves a strong impression that the adenomata are the outcome of anomalous repair and regeneration processes following prolonged inflammation in the lung. Not only is it possible to trace the stages of development of a tumour in the chronic foci of inflammation and collapse, but the striking difference in the incidence of pneumonia with urethane as compared with the other anaesthetic substances used is highly suggestive. Since this work was done, Larsen (1946) has also shown that urethane is so far unique amongst narcotic compounds tested ; he used a larger selection of such substances than was employed here, but without finding any other which produced a significant increase in pulmonary adenomata.

In their original communication Nettleship and Henshaw (1943) did not appear to be impressed with the evidence of inflammation. They admit that inflammatory cells are present, but state that they are few in number. They found that conditions such as bronchiectasis, purulent bronchitis and acute pneumonia were distributed alike among the experimental and control animals. They are emphatic that lung tumours and pneumonia were never seen together, though they occurred apart from each other. It is possible that this difference of interpretation may depend on what is meant by the term pneumonia. With massive or lobar consolidation, as opposed to collapse inflammation, adenomata have also been absent in the present mice. This is not altogether surprising, because if the adenomata do arise as an end result of chronic inflammation, they would not be expected in the lobar consolidation type of lesion, which presumably would either be fatal or undergo fairly rapid resolution. Nevertheless, Nettleship and Henshaw remark that the negative correlation between pneumonia and adenomata may be significant.

They also raise the question of the actual effective agent being a virus, and this thesis is developed by Henshaw and Meyer (1944), who state that certain observations, " together with the findings that little or no tissue injury precedes the appearance of lung tumours, make it plain that urethane carcinogenesis has characteristics usually associated only with viruses, specific tissue action, remoteness of induction site, and absence of precancerous injury." As has been seen, the present author is not satisfied as to the validity of some of their premises. Further, there is no evidence of primary degenerative changes in the bronchial epithelium, such as have been described by Straub (1937) for influenza virus ; the participation of the bronchial epithelium in the process is late, and takes the form of proliferation into an already damaged tissue.

### SUMMARY.

The findings of Nettleship and Henshaw (1943) that treatment with urethane gives rise to a greatly increased incidence of pulmonary adenomata in mice have been confirmed. The effect has been found in all mice tested, though some strains are more susceptible than others. Various other anaesthetic substances gave negative results.

The immediate effect of urethane is to set up chronic inflammation in the lungs, and the adenomata appear to arise as the result of further changes in the inflammatory lesions; in some cases there is evidence that they are derived from proliferation of the bronchial epithelium. Not all the macroscopic nodules are tumours; some regress if treatment is discontinued. The present series contained one malignant tumour, the histology of which was different from that of the adenomata.

### REFERENCES.

HENSHAW, P. S., AND MEYER, H. L.—(1944) *J. nat. Cancer Inst.*, **4**, 523.—(1945) *Ibid.*, **5**, 415.
HOYLE, L., AND ORR, J. W.—(1945) *J. Path. Bact.*, **47**, 441.
LARSEN, C. D.—(1946) *J. nat. Cancer Inst.*, **7**, 5.
NETTLESHIP, A., AND HENSHAW, P. S.—(1943) *Ibid.*, **4**, 309.
STRAUB, M.—(1937) *J. Path. Bact.*, **50**, 31.